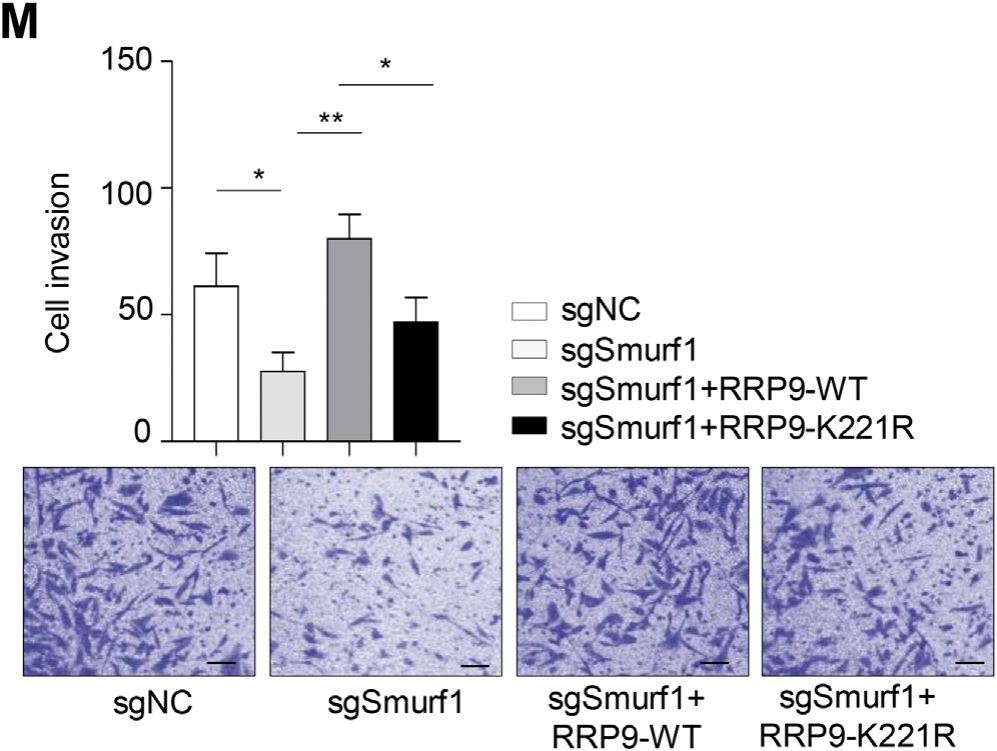# Correction: Neddylation modification of the U3 snoRNA-binding protein RRP9 by Smurf1 promotes tumorigenesis

**DOI:** 10.1016/j.jbc.2022.102567

**Published:** 2022-10-18

**Authors:** Meng-ge Du, Fan Liu, Yan Chang, Shuai Tong, Wei Liu, Yu-jiao Chen, Ping Xie

The original versions of Figures 6L and 6M were incorrect. The Figure 6L image sgSmurf1+RRP9-WT was inadvertently taken from Figure 5D (sgRRP9+RRP9-WT image). Figure 6M image sgSmurf1+RRP9-WT was unintentionally misused and Figure 6M graph had mislabeled Y-axis coordinates. The statistics of Figure 6M were reset and the graph labelling was corrected. The correct Figures 6L and 6M are now provided. These corrections do not change the interpretation of the results or the conclusions. The authors apologize for those mistakes.

**Figure:**
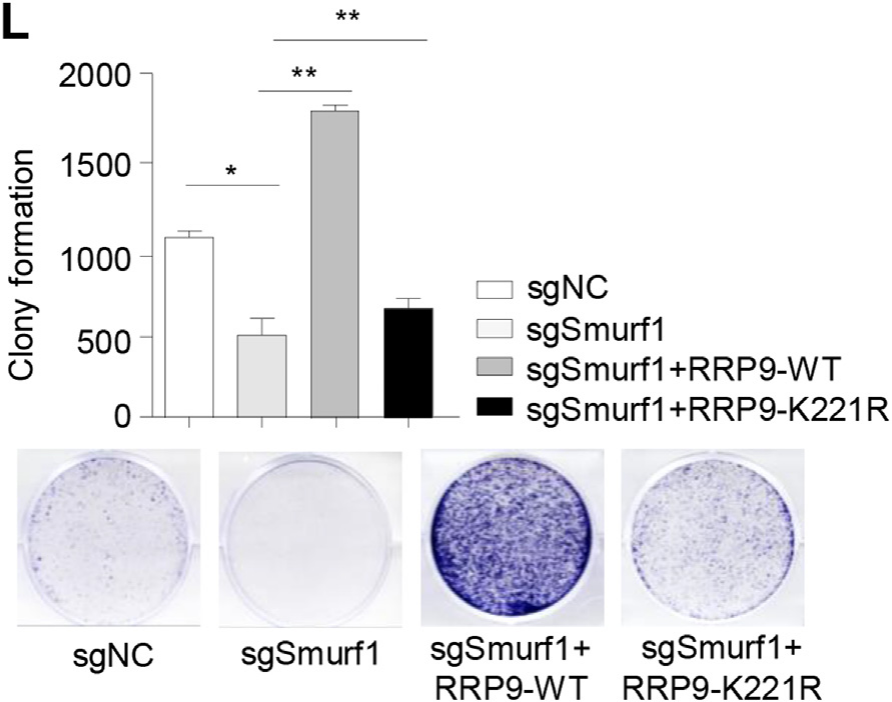

**Figure:**